# Effect of Shot Peening on the Strength and Corrosion Properties of 6082-T651 Aluminium Alloy

**DOI:** 10.3390/ma16144976

**Published:** 2023-07-12

**Authors:** Dunja Ravnikar, Roman Šturm, Sebastjan Žagar

**Affiliations:** Faculty of Mechanical Engineering, University of Ljubljana, Askerceva 6, 1000 Ljubljana, Slovenia; dunja.ravnikar@fs.uni-lj.si (D.R.); roman.sturm@fs.uni-lj.si (R.Š.)

**Keywords:** shot peening, aluminium alloy, surface roughness, Vickers hardness, residual stresses, corrosion resistance

## Abstract

This paper investigated the effect of shot peening on the strength and corrosion properties of 6082-T651 aluminium alloy. The microstructure, surface roughness, microhardness, residual stresses, and corrosion behaviour were investigated and compared with those of untreated aluminium alloy. Cracks and delaminations in the surface layer could only be seen on the treated specimens at a working pressure of 4 bar and 8 bar, while no such effect was observed at a working pressure of 1.6 bar. The surface roughness was increased more than 15 times after shot peening at a working pressure of 8 bar. Shot peening resulted in an increase in microhardness of 27% and a deeper layer with higher compressive residual stresses compared to the untreated specimens. All treated specimens exhibited improved corrosion resistance with a lower degree of anodic dissolution. The current density of the treated specimen with the lowest surface roughness was more than two-fold lower than that of the untreated specimen.

## 1. Introduction

Aluminium and its alloys are widely used in many areas of industry, such as the automotive, aerospace, shipping, sports, electrical, machinery, and petroleum sectors, primarily because of their light weight, good weight-to-strength ratio, excellent electrical and thermal conductivity, low cost, and corrosion resistance in non-aggressive environments [1,2,3,4,5,6]. Although a thin oxide film that naturally forms on the surface of an aluminium alloy can partially prevent corrosion attack, the presence of Cl^−^, F^−^, I^−^, and Br^−^ ions in aqueous solutions causes the poor corrosion resistance of aluminium alloys [1,4,5]. In addition, their poor fatigue resistance, low surface hardness, and poor wear resistance also make them inadequate for many requirements [3,7,8]. Therefore, the additional surface protection of aluminium alloys is often essential and is usually achieved by various coatings applied on aluminium alloys [1,2,5] or other surface processing techniques, such as different methods of shot peening [4,6,7,8,9,10,11,12,13,14,15], cavitation peening [16], and surface remelting [3,17].

Shot peening (SP) is an effective a surface treatment method used to improve the surface life and durability of components exposed to both general fatigue and fretting fatigue [18]. SP involves bombarding the surface of a material with small, spherical particles. These shot particles, typically made of materials like steel, ceramic, or glass beads, are propelled at high velocities using compressed air, pressurised water, ultrasonic energy, or centrifugal force. The impact of the shots creates compressive residual stresses on the surface of the component [19]. By introducing compressive stresses, SP prevents crack initiation and propagation, improving the fatigue resistance of the material. Additionally, shot peening refines the grain structure of the material, resulting in improved fatigue strength and resistance to stress corrosion cracking. Due to the simplicity of the process and the fact that there is no heat exposure, SP can be applied to any complex and intrinsic shape [7].

Efe et al. [7] studied severe SP with an Almen intensity of 20 A on an AA7075 aluminium alloy. They reported that two gradient layers were formed: a deformed layer and a nanocrystalline layer. The microhardness was improved by 30% on the treated surface but dropped to less than 10% after 600 µm depth. An improvement in fatigue behaviour was also observed after shot peening, although the roughness increased significantly. In addition, the friction coefficient of the treated specimens was generally lower, and limited loads and excessive sliding paths could have had a negative effect on the wear performance of SP, while increasing sliding paths caused the disappearance of the influence of shot peening. The comparison between conventional SP and micro-SP was investigated by Su et al. [9]. They concluded that micro-shot peening improved the fatigue properties of bare specimens over conventional shot peening because it inhibited crack initiation, while for micro-arc oxidised alloys with stringent corrosion and abrasion resistance requirements, conventional shot peening improved fatigue properties better due to its inhibitory effect on crack propagation. However, conventional shot peening resulted in a deeper affected layer with higher compressive residual stresses, but on the other hand, the surface roughness increased due to large-size particles at a high velocity, which inevitably weakened the improvement of fatigue properties. Wang et al. [10] applied a novel coupled constitutive model to study the effect of different shot sizes on the microstructure of a 2060 Al-Li alloy and reported that a larger shot size improved the depth of the dislocation density layer.

Sun et al. [11] investigated ultrasonic shot-peened AA7150 and reported that the corrosion resistance of treated specimens in the environments was improved in comparison with untreated specimens. The improved local corrosion resistance was due to the formation of equiaxed nano-grains and the homogenisation of the microstructure on the treated layer. Hao et al. [14] also found that low SP prevented the formation and growth of corrosion pits, mainly due to grain refinement. Trdan et al. [4] showed that femtosecond laser peening improved the corrosion resistance of AA2024-T3 due to compressive residual stress. Despite the improvement in corrosion resistance, Qiao et al. [6] investigated the corrosion resistance of 2024 aluminium alloy in a salt spray environment. They reported that the corrosion resistance of 2024 aluminium alloy decreased after SP, with pitting corrosion occurring first in the crater lap zone and became more severe with increasing salt spray time.

Shot peening is often reported to increase the surface hardness, compressive residual stress, and associated high-density dislocation structures, improving fatigue and wear properties and extending the machine part’s life. On the other hand, the increased surface roughness caused by SP can lead to pitting corrosion. Therefore, the aim of this study was to investigate the effects of conventional shot peening with different SP parameters on strength and corrosion properties. Microhardness and residual stresses tests were performed to evaluate the strengthening properties. In addition, corrosion properties were investigated using open-circuit potential and cyclic potential scans.

## 2. Experimental Method

### 2.1. Specimen Preparation and Shot Peening

The substrate used was a commercially available 6082-T651 (AlSi1MgMn) aluminium alloy with the chemical composition given in Table 1. The aluminium alloy used was obtained in the state T651. This meant that it was homogenised at a temperature of 540 °C, then cold hardened with 1–3% deformation, and finally artificially aged or precipitation hardened at a temperature of 160 °C for 10 h. The mechanical properties of the 6082-T651 aluminium alloy are listed in Table 2.

Specimens treated by the conventional shot peening process with hard particles mixed in pressurised air were hardened to various degrees. The hard shots flying out of the nozzle covered a larger area of the workpiece, being most concentrated in the core of the jet. The overlap of the individual hard shots was defined by the mass flow to the surface and travel velocity.

SP was carried out with different working pressures, mass flows, and Almen intensities to achieve different degrees of hardening. The Almen intensity is a parameter used in shot peening to quantify the intensity of the shot peening process. The values of the Almen density indicate the amount of strip deflection (arc height) that results from the shot peening process. The greater the deflection or arc height, the higher the Almen intensity and the more intense the shot peening treatment.

The working medium was the same for all four settings of the working machine, which had the designation S17056 HRC. On the label, the letter S (steel) stood for the type of particles, and the number 170 for the diameter of the particles, which in our case was 0.4 mm. A constant parameter in the surface treatment was also the distance between the nozzle of the processing machine and the surface of the workpiece.

The data on the parameters of SP are summarised in Table 3. The designation 6082 represents an untreated specimen, while 6082/3, 6082/7, 6082/12, and 6082/16 represent treated specimens with different working pressures, mass flows, and Almen intensities.

### 2.2. Microstructural and Mechanical Analysis

To gain an insight into the microstructure, it was necessary to properly cross-sect the specimen and prepare the specimens for a metallographic analysis. Specimens were ground with 320- to 800-grit SiC paper and polished with colloidal silica of 3 μm and 1 μm. To make the microstructure visible, an etching solution of 5% HF + 95% water was used. Microstructural observations were performed on the cross-sections of SP specimens using an Olympus SZX10 optical microscope. By analysing the microstructure, we tried to determine the effects of particle collisions on the changes in the hardened surface layer. Distance measurements between precipitates were conducted using the UTHSCSA Image Tool program v3.0 (IT).

A Surtonic 3+ (Taylor/Hobson Peumo) was used to measure surface roughness, and TalyProfile Silver v7.4 software was used to calculate the roughness parameters, i.e., the mean arithmetic roughness (*R*_a_) and the mean roughness depth (*R*_z_). All measurements with a length of 8 mm were conducted in different directions at the edge and in the center of the specimen with 10 repetitions each. The average *R*_a_, average *R*_z_, and standard deviation was were calculated.

Microhardness was measured using a Vickers Leith tester (Wetzlar, Germany) at a load of 200 g and 15 s indentation time. Measurements were taken in increments of 25 μm to a depth of 350 μm and repeated at six measurement points. Subsequently, the average microhardness with standard deviation was then calculated.

The hole-drilling method according to the ASTM E837 standard [20] was used to measure the residual stresses. CEA-06-062UL-120 resistive rosettes manufactured by Vishay Precision Group, Malvern, PA, USA, and a 1.6 mm diameter drill were used for the measurement. ReStress for WindowsTM v1.07 software (Vishay Group Inc.) was used to measure deformation and motion and to calculate the residual stress profiles using the integral method.

### 2.3. Corrosion Analysis

The electrochemical corrosion tests were evaluated using VoltaLab 10 PGZ 100 equipment (Radiometer Analytical SAS, Villeurbanne, France) with VoltaMaster v4 software according to the ASTM G5-14 standard [21]. A classical three-electrode cell was used for the corrosion tests. A saturated calomel electrode (SCE) served as the reference electrode, and the untreated and shot-peened specimens embedded in PAR Teflon holders served as the working electrode. For the study, a naturally aerated (*pH* = 7.5 ± 0.2, *T* = 22.6 ± 0.3 °C) 0.5 M NaCl solution was chosen, freshly prepared with deionised water from laboratory-grade NaCl (Sigma Aldrich, St. Louis, MO, USA) before each experiment. Before each corrosion test, all specimens were cleaned and degreased in an ultrasonic bath containing ethanol and then in deionised water for 3 min each. To ensure the reproducibility of the results, each test was performed twice in the same manner. For *E*_corr_, *E*_sw_, *E*_prot_, and *i*_corr_, the average value and standard deviation were calculated.

The open-circuit potential (OCP) measurement was maintained for 60 min to stabilise the surface, and then the corrosion potential (*E*_corr_) was determined at the end of the stabilisation process. After the OCP measurement, a cyclic polarisation test (CP) was performed in the anodic direction of −200 mV with respect to the OCP at a rate of 1 mV/s. The polarisation direction was reversed to the cathodic direction at the switching potential (*E*_sw_) with the potential reaching a limited threshold of 1 mA/cm^2^. The corrosion potential (*E*_corr_) and corrosion current density (*i*_corr_) were determined using the Tafel extrapolation method [22]. In addition, the switching potential (*E*_sw_) and the potential at which the pits became repassive during backward scanning (*E*_prot_) were also determined. The protective effect (*P*_ef_) to obtain quantitative information was calculated according to the following Equation (1) [1,5]:

(1) $$P_{\mathrm{EF}}\left(\%\right)=\frac{i_{\mathrm{corr},0}-i_{\mathrm{corr},N}}{i_{\mathrm{corr},0}}$$

where *i*_corr,0_ and *i*_corr,N_ represent the corrosion current densities of the untreated and shot-peened aluminium specimens, respectively.

## 3. Results and Discussion

### 3.1. Microstructural and Surface Roughness Analysis

**Figure 1:**
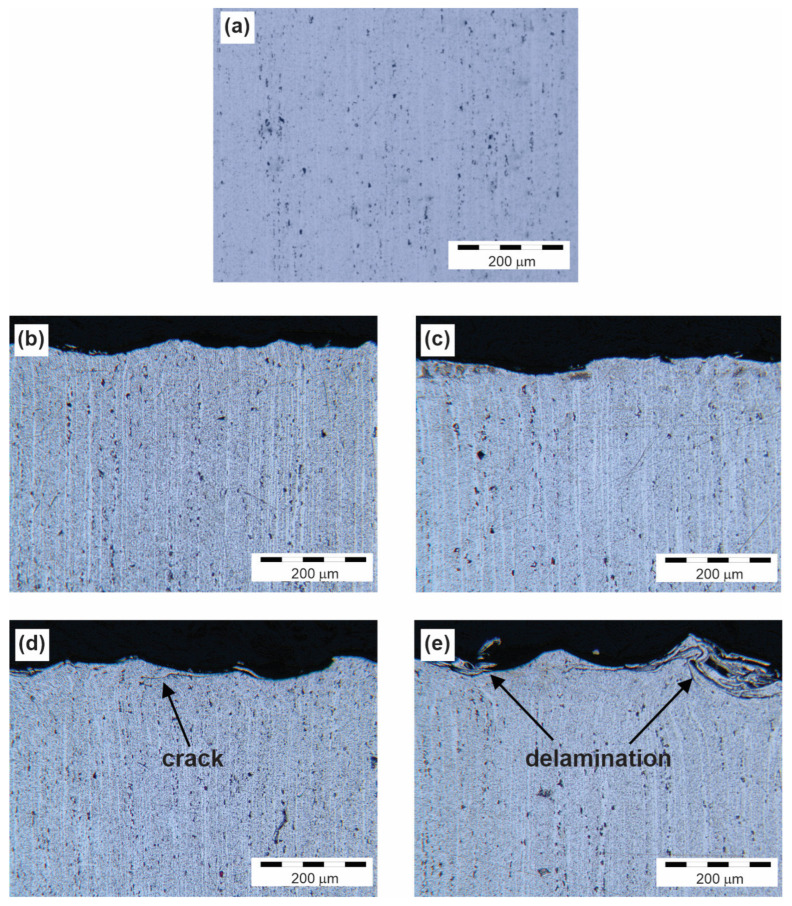
The cross-sectional microstructure of (**a**) untreated specimens and treated specimens (**b**) 6082/3, (**c**) 6082/7, (**d**) 6082/12, and (**e**) 6082/16.

Figure 1 shows the microstructures of the untreated (Figure 1a) and treated specimens (Figure 1b–e) for each condition individually, revealing hardened precipitates and surface grooves at different working pressures. The microstructural images were taken directly below the surface.

The microstructure of the specimens treated under the hardest conditions in Figure 1c,d showed cracks and delaminations in the surface layer, which represented the negative effects of hardening. No such defects could be seen in the microstructure of the specimens hardened at a working pressure of 1.6 bar (Figure 1b,c). The same surface defects were also found by Trško et al. [13] in their study.

**Figure 2:**
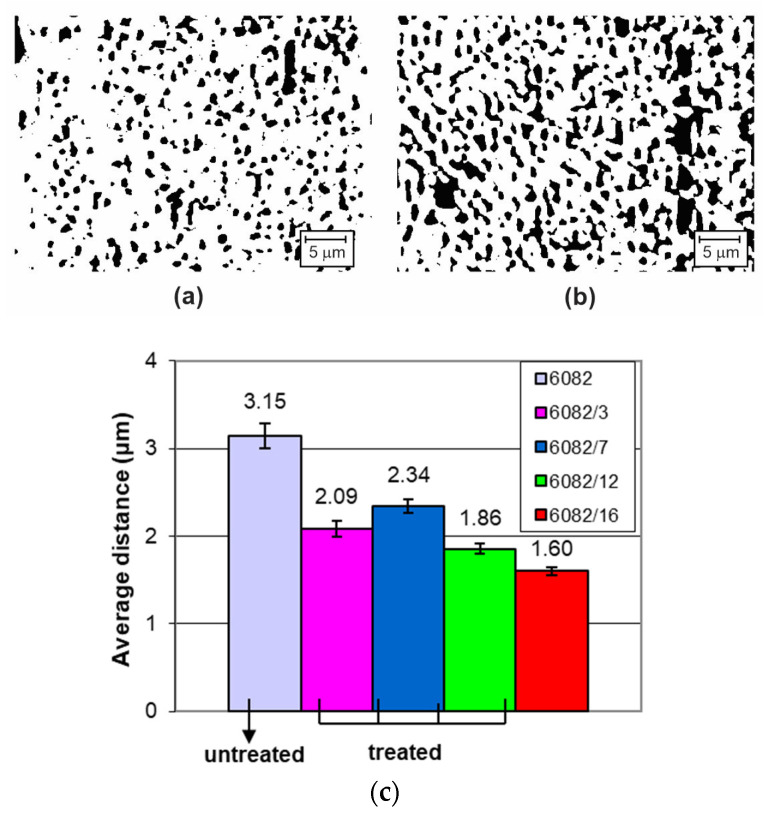
The number of precipitated phases for (**a**) the untreated 6082 specimen and (**b**) the treated 6082/16 specimen; (**c**) average distances between individual precipitates.

SP can have an impact on the precipitates in aluminium alloys. Figure 2a,b shows the number and distribution of precipitates in the high-magnification microstructure of the untreated specimen (Figure 2a) and specimen treated at a working pressure of 8 bar (Figure 2b), while Figure 2c presents a bar graph with the average values of the distances between individual precipitates. The distances between the precipitates were measured on the image of the microstructure. The measurement area was approximately 45 μm × 35 μm in size and was located approximately 45 μm below the surface in all specimens.

A greater amount of precipitation was observed in the treated aluminium alloys. Plastic deformation due to cold hardening with hard shots brought the precipitates together. Moreover, the distance between the precipitates became slightly shorter under stricter SP conditions. The greater the kinetic energy of the shots, the greater the plastic deformation that occurred and the shorter the distances between the precipitates present.

Precipitates act as obstacles to dislocation movement, impeding its movement and promoting strain hardening during the peening process. This can lead to a more pronounced strengthening effect and higher dislocation densities in the material. The effect of dislocation densification was confirmed by an additional measurement of the microhardness above the hardened layer.

SP is known to cause surface roughness on the treated material. The surface roughness after SP treatment depends on the hardness of the aluminium alloy, the hardness of the shot particles, and the kinetic energy of the particles falling on the surface.

**Figure 3:**
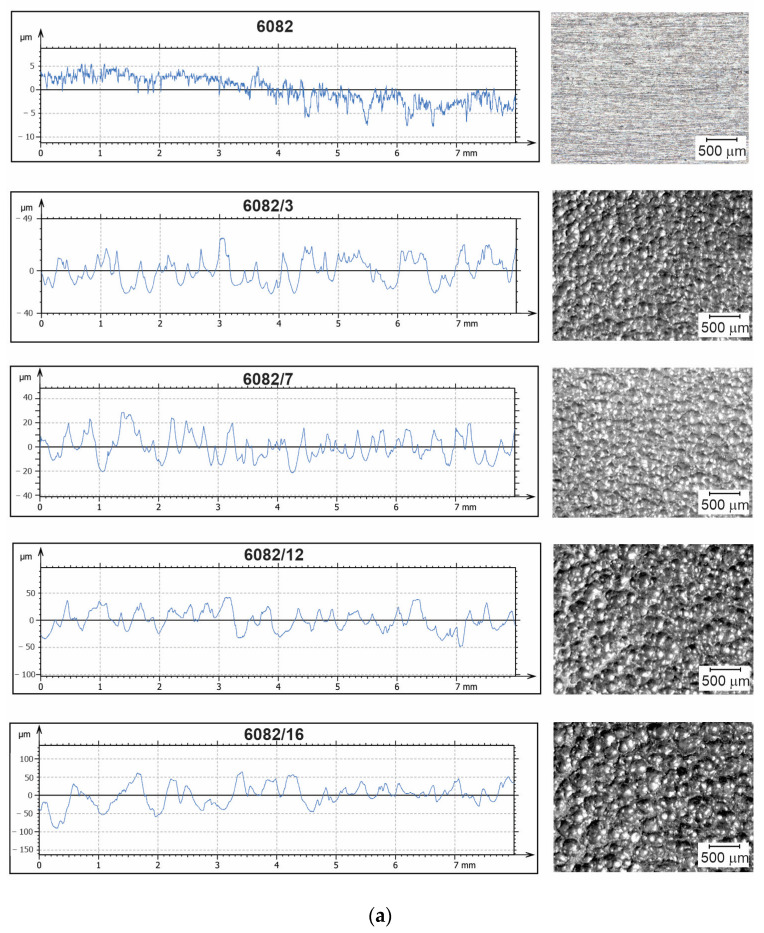

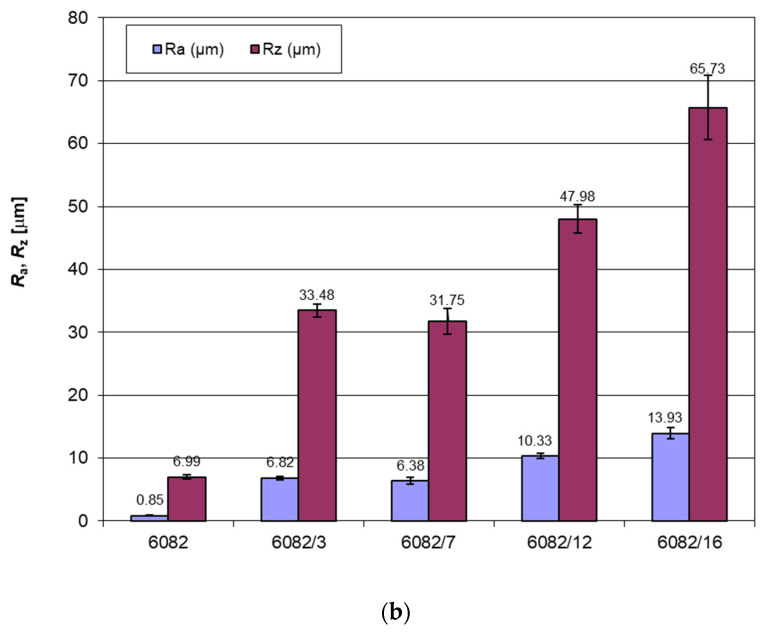
(**a**) Surface roughness profiles and surface topography, (**b**) arithmetic mean roughness (*R*_a_) and mean roughness depth (*R*_z_) before and after treatment.

Figure 3a shows a surface roughness profile measurement and surface topography for untreated and treated specimens. After bombarding the surface material with steel particles, dimples were created on the specimens. The effect of the impact SP treatment on the topography can be seen with the naked eye on our specimens. We can visually judge that the specimens treated with a higher working pressure have greater roughness than the specimens treated with a lower working pressure. From the surface roughness profiles, the quantitative values can be obtained to describe surface roughness characteristics.

Figure 3b shows, in the form of bar charts, the mean values of the arithmetic mean roughness (*R*_a_) and the mean values of the roughness depth (*R*_z_), which increased several times for all specimens compared to the untreated specimens. The *R*_a_ and *R*_z_ values of the untreated aluminium alloy were 0.85 ± 0.05 μm and 6.99 ± 0.33 μm, respectively.

From the surface roughness data, it follows that as the working pressure increased, the roughness of the hardened surface also increased. For specimens treated with the same working pressure of 1.6 bar but with different mass flows, no significant differences were found in the calculated surface roughness characteristics. The *R*_a_ and *R*_z_ values even slightly decreased (6.82 ± 0.28 μm to 6.38 ± 0.50 μm and 33.48 ± 1.01 μm to 31.75 ± 2.08 μm) when the mass flow rate was increased from 1 kg/min to 1.5 kg/min at the same pressure. For the specimens treated at 4 bar and 8 bar working pressures, which meant that the particles had a higher kinetic energy, it was found that the roughness increased with an increased working pressure. The *R*_a_ and *R*_z_ values of the treated aluminium alloy at 4 bar were 10.33 ± 0.43 μm and 47.98 ± 2.26 μm, respectively, and for the alloy treated at 8 bar, the *R*_a_ and *R*_z_ values were 13.93 ± 0.91 μm and 65.73 ± 5.15 μm, respectively. The arithmetic mean roughness was increased more than 15 times. An increase in roughness during hardening is characteristic of softer materials, including the tested EN AW 6082 aluminium alloy [23].

### 3.2. Microhardness and Residual Stress Analysis

The high-velocity impact of the shot particles induces plastic deformation on the surface, leading to dislocation movement and rearrangement within the material. This results in an increase in the microhardness of the material. Furthermore, through SP, the grains of the material are refined [10,12]. Grain refinement results in an increase in hardness according to the Hall–Petch relationship [24], where the microhardness of the material increases with a finer grain size [25,26].

**Figure 4:**
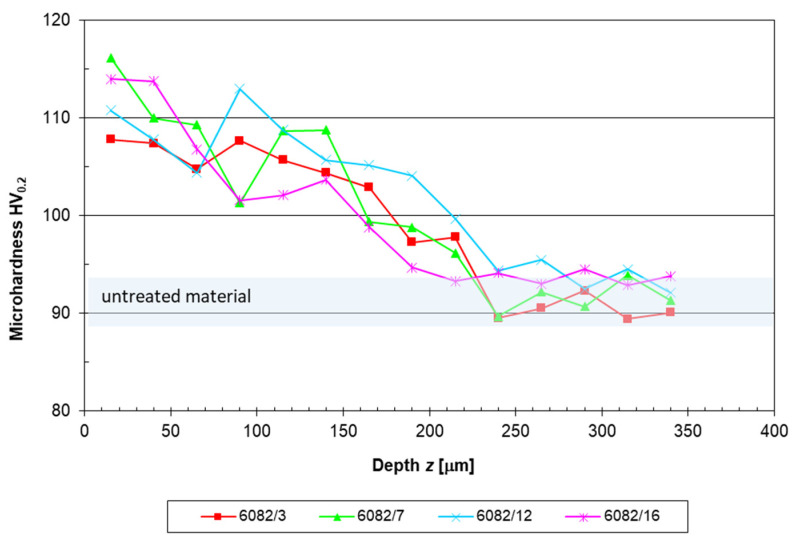
Trough-depth microhardness profiles of untreated and shot-peened specimens (insert: near-surface microhardness HV_0.2_).

Figure 4 presents the microhardness profiles of the SP-treated and untreated specimens. It can be clearly seen that the microhardness was generally higher near the treated surface, while the microhardness decreased to the value of the base material with an increasing distance from the surface. The microhardness of the untreated material was 91 ± 3 HV_0.2_. The largest increase in microhardness near the surface due to SP was observed in specimen 6082/7 with a value of 116 ± 3 HV_0.2_, which exhibited a 27% increase in microhardness compared to the untreated material. The mean microhardness near the surface of the 6082/3, 6082/12, and 6082/16 specimens was 108 ± 4 HV_0.2_, 111 ± 5 HV_0.2_, and 114 ± 5 HV_0.2_, respectively. The differences between the microhardness values with standard deviations were very small, despite repetitions at random locations 25 µm below the surface. Therefore, we could not determine the effects of different SP parameters on the surface microhardness. Shot peening, however, successfully improved microhardness near the surface.

**Figure 5:**
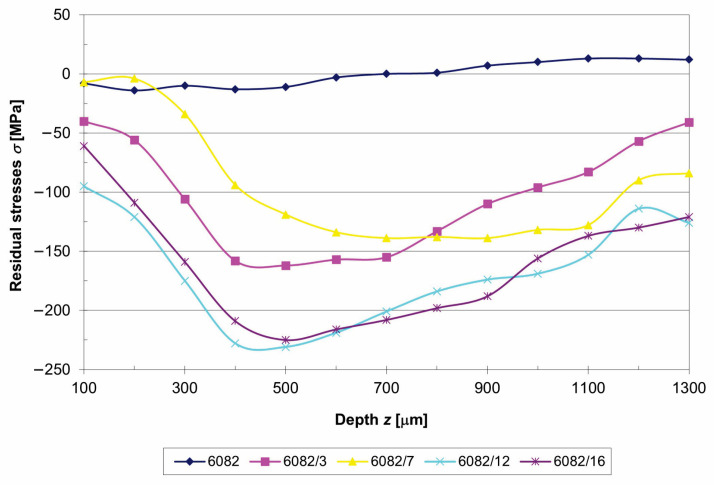
Residual stress profiles of untreated and shot-peened specimens.

Figure 5 shows the residual stresses for the treated and untreaded aluminium alloy EN AW 6082. The untreated specimen had a maximum compressive residual stress of −14 MPa at a 200 μm depth. Below a depth of 600 μm, the residual stresses became tensile.

The residual stresses for treated specimens initially increased or escalated to the highest value, at a depth between 400 μm and 700 μm, regardless of the hardening conditions. Differences were found in the hardening depth and in the value of compressive stresses. For specimen 6082/7, the maximum compressive residual stress of −139 MPa was reached at a depth of 700 μm. The maximum value of the compressive residual stresses for specimen 6082/3 was −162 MPa at a depth of 500 μm. When the working pressure was increased to 8 bar (6082/16 specimen), the compressive residual stress profiles increased more rapidly with the increasing depth of the hardened layer, reaching their maximum compressive stress of −225 MPa at a depth of 500 μm. The highest maximum compressive residual stresses of −231 MPa at a depth of 500 μm were found in specimen 6082/12. The largest difference in residual stresses was found between specimens 6082/7 and 6082/12 and was 112 MPa at a depth of 500 μm.

The applied compressive residual stresses reached 60–70% of the yield strength. Exactly these magnitudes were reached under the hardest conditions at working pressures of 4 bar and 8 bar.

### 3.3. Corrosion Analysis

The open-circuit potential (OCP) is the electrochemical potential at which a system is in thermodynamic equilibrium, representing the balance between cathodic and anodic reactions; it can be used to evaluate the tendency of a material to participate in an electrochemical reaction [27]. In general, the more negative the value of the OCP, the greater the material’s tendency to participate in electrochemical corrosion reactions [2].

**Figure 6:**
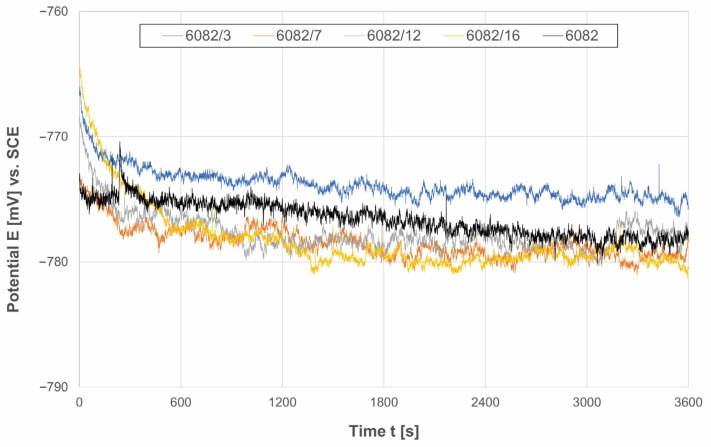
Open-circuit potential of untreated and treated 6082 aluminium alloy in 0.5 M NaCl solution.

The OCP curves of the shot-treated and untreated 6082 aluminium alloy in a 0.5 M NaCl solution are shown in Figure 6.

As can be seen from the potential–time behaviour, all specimens showed a similar response during OCP testing with similar *E*_corr_ values at the end of the stabilisation process (from −776 ± 11 mV_SCE_ to −781 ± 9 mV_SCE_ for shot-peened specimens and −778 ± 13 mV_SCE_ for the untreated specimen). When the specimens were immersed in an NaCl solution, all curves exhibited a slow potential decrease with slight fluctuations. The fluctuations (noise) in potential may have resulted in metastable pitting events, including the dissolution, breakdown, or rupture and partial repair of the surface oxide layer [5,28].

**Figure 7:**
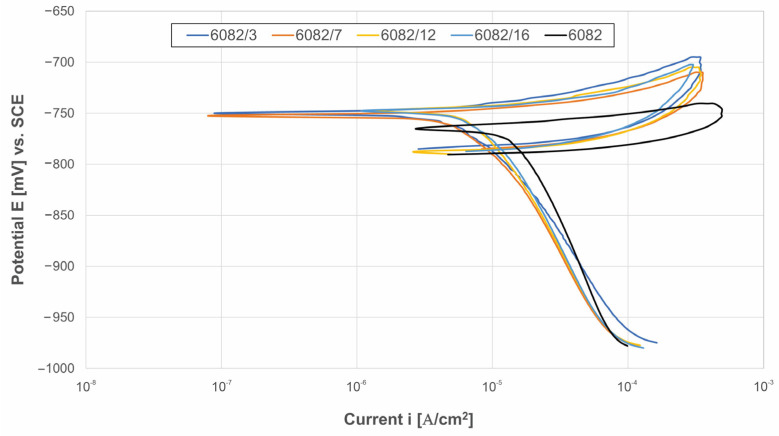
Cyclic polarisation curves of the untreated and treated 6082 aluminium alloy in a 0.5 M NaCl solution.

Following the OCP test, cyclic potentiodynamic (CP) scanning was recorded in a 0.5 M NaCl solution. The CP method allows the quantitative evaluation of the pitting resistance on the surface of aluminium alloys. In summary, an increased corrosion potential (*E*_corr_) indicates a lower corrosion tendency, while a decreased corrosion current density (*i*_corr_) is associated with a lower corrosion rate [1]. The polarisation curves of the untreated and shot-peened specimens are shown in Figure 7, while the electrochemical parameters are listed in Table 4.

The CP curves of the shot-peened specimens and the CP curve of the untreated specimen had a similar shape regardless of the specimen, where the cathodic curve intersected with the anodic scan, confirming that all tested specimens were susceptible to pitting corrosion. However, there was a significant difference between the treated and untreated specimens. The CP curve of the untreated specimen presented a sharp increase in current during anodic polarisation, while partial passivation was observed after the shot-peening process, as the potential increased slightly with the increasing current density.

Furthermore, the experimental results confirmed that the shot peening significantly affected the important corrosion parameters such as the potential (*E*_corr_) and corrosion current density (*i*_corr_). The most negative *E*_corr_ value (−766 ± 15 mV_SCE_) was observed in the untreated specimen, while the noblest corrosion potential was exhibited by the shot-peened specimens 6082/12 and 6082/16 (−748 ± 14 mV_SCE_ and −748 ± 11 mV_SCE_, respectively). The current density *i*_corr_ of 6082/3 with the lowest surface roughness among the treated specimens was more than two-times lower than the untreated specimen (9.7 ± 0.7 μAcm^−2^ vs. 22.5 ± 2.9 μAcm^−2^). The current density of the treated specimens increased with surface roughness, and even specimen 6082/16 with the highest roughness had a current density 1.8 times lower than that of the untreated specimen (12.2 ± 1.1 μAcm^−2^ vs. 22.5 ± 2.9 μAcm^−2^). Clearly, all treated specimens improved the corrosion resistance of the Al 6082-T651 alloy. In addition, the results of the protective effect showed a positive effect of shot peening, with the highest value being 57%, notwithstanding the higher roughness of the treated specimens compared to the untreated specimen. The key parameters used to describe the resistance to pitting corrosion in a chloride-containing solution are the absolute differences between *E*_sw_ and *E*_corr_, and *E*_corr_ and *E*_prot_ [5]. A larger value of the absolute difference between *E*_sw_ and *E*_corr_ signifies a reduced degree of anodic dissolution of the material, whereas a smaller value of the absolute difference between *E*_corr_ and *E*_prot_ indicates an enhanced ability for material repassivation. By analysing the tendence of Δ*E*, it could be confirmed that all treated specimens exhibited a decreased degree of anodic dissolution, while the untreated substrate demonstrated a faster and better ability for repassivation.

Considering the CP result, it could be concluded that shot peening improved the corrosion resistance in comparison with the untreated aluminium alloy. These results were in agreement with those of Sue et al. [11], who showed that the corrosion resistance of AA7150 was significantly improved by ultrasonic shot peening in a 57 g/L NaCl + 10 mL/L H_2_O_2_ solution compared to its untreated counterpart, which was attributed to the formation of equiaxed nanograins and the homogenisation of the microstructure on the peened layer. Furthermore, Trdan et al. [4] reported in their study that the CP results confirmed a four-times lower corrosion current density for the specimen peened with a femtosecond laser, assuming that the surface roughness was more than two times lower after femtosecond laser peening compared to conventional shot peening.

**Figure 8:**
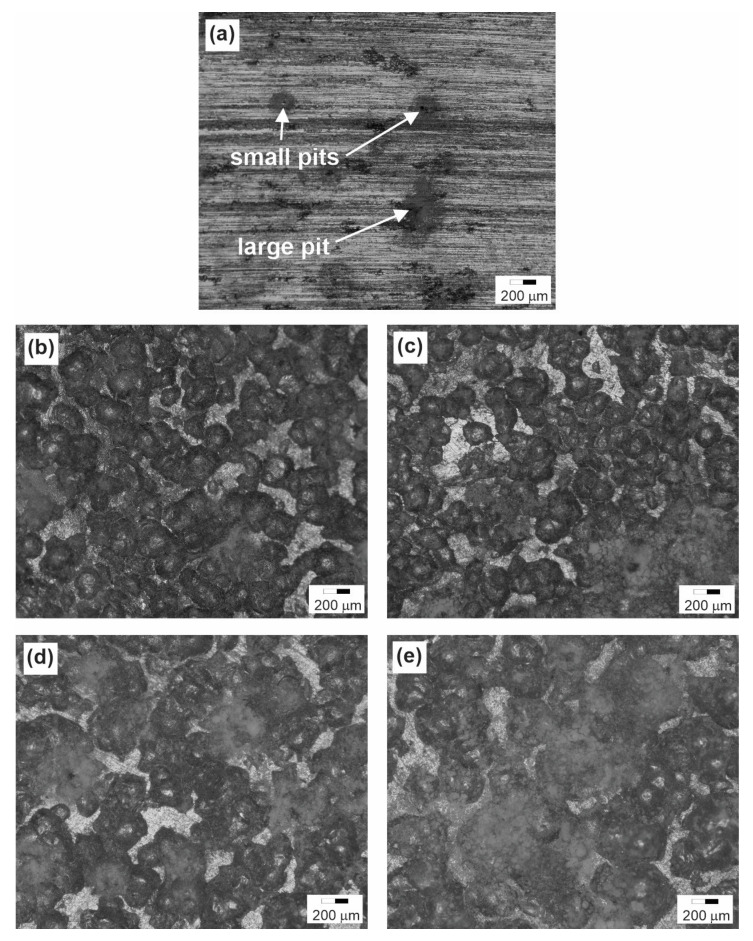
Surface observations after CP testing of (**a**) untreated specimen and treated specimens (**b**) 6082/3, (**c**) 6082/7, (**d**) 6082/12, and (**e**) 6082/16.

Figure 8 shows the surface of untreated and SP-treated specimens after CP corrosion tests. In the untreated specimen, the widespread attack of pitting corrosion over the entire surface was observed. The corrosion attack was weaker for the treated specimens but increased with an increasing working pressure.

However, the effects of surface roughness [28,29] and compressive residual stresses [4,10,30] play an important role in corrosion-resistant materials. Rough surfaces can provide more sites for corrosion initiation and accelerate the corrosion process. Smoother surfaces, on the other hand, have better corrosion resistance because there are fewer crevices and preferential sites for corrosion to develop. Compressive residual stresses have a positive impact on corrosion resistance, because they close cracks, reduce crack propagation, and enhance the material’s resistance to corrosion-induced cracks. Furthermore, smoother surfaces with compressive residual stresses exhibit increased resistance to localised corrosion, because the compressive stresses counteract the detrimental effect of surface roughness. This combination can effectively reduce the likelihood of corrosion initiation and progression.

## 4. Conclusions

Shot peening was carried out on an EN AW 6082-T651 aluminium alloy, which effectively improved residual stresses, microhardness, and corrosion resistance. The following conclusions could be drawn from the results obtained:

1. The shot peening process was successfully implemented with a working pressure of 1.6 bar without any defects. At a working pressure of 4 bar and 8 bar, cracks and delaminations appeared in the surface layer, which represented the negative effects of hardening.
2. An increased number of precipitates was observed in treated aluminium alloys, where plastic deformation caused by SP brought the precipitates closer together.
3. SP increased the surface roughness, which increased with more intense SP conditions. At a working pressure of 8 bar, the *R*_a_ was increased 15.3 times.
4. The shot peening process proved to be an effective method of producing high compressive residual stresses and increased hardness in the treated surface layer. Microhardness increased by up to 27% compared to the untreated specimen. The compressive residual stresses were deeper and higher than in the untreated aluminium alloy. The untreated specimen had a maximum compressive residual stress of −14 MPa at a depth of 200 μm, while the treated specimen at a working pressure of 4 bar reached the maximum compressive stress of −231 MPa at a depth of 500 μm.
5. All treated specimens improved the corrosion resistance of the EN AW 6082-T651 aluminium alloy, although the shot peening caused greater surface roughness. However, all treated specimens exhibited compressive residual stresses, which had a beneficial effect on corrosion resistance.
6. The untreated aluminium alloy revealed no passive behaviour, while the treated specimens exhibited partial passivation, and all treated specimens had a much lower corrosion current density (up to 2.3 times lower compared to the untreated aluminium alloy) and a protective effect of up to 57%.
7. All treated specimens exhibited a lower degree of anodic dissolution, while the untreated specimen offered a better and faster ability to repassivate.

## Figures and Tables

**Table 1:** Chemical composition of EN AW-6082.

Si	Mg	Mn	Fe	Cr	Zn	Ti	Cu
(wt.%)	(wt.%)	(wt.%)	(wt.%)	(wt.%)	(wt.%)	(wt.%)	(wt.%)
0.87	0.72	0.42	0.35	0.02	0.04	0.03	0.05

**Table 2:** The mechanical properties of EN AW-6082.

Condition	*R*m	*R*_p0.2_	*A*	HV_0.2_
	(MPa)	(MPa)	(%)	
T651	326	298	12	89

**Table 3:** The parameters of shot peening.

Specimen Designation	Working Pressure	Mass Flow	Almen Intensity	Nozzle Distance	Type of Working Medium
	*p* (bar)	*ṁ* (kg/min)	*I* (mmA)	*y* (mm)	(/)
6082	/	/	/	/	/
6082/3	1.6	1.0	0.25	4	S170HRC56
6082/7	1.6	1.5	0.31	4	S170HRC56
6082/12	4	1.6	0.53	4	S170HRC56
6082/16	8	1.5	0.7	4	S170HRC56

**Table 4:** Electrochemical parameters.

	6082	6082/3	6082/7	6082/12	6082/16
*R*_a_ (m)	0.85 ± 0.05	6.82 ± 0.28	6.38 ± 0.50	10.33 ± 0.43	13.93 ± 0.91
*E*_corr_ (mVSCE)	−766 ± 15	−750 ± 9	−753 ± 13	−748 ± 14	−748 ± 11
*E*_sw_ (mVSCE)	−741 ± 10	−700 ± 11	−715 ± 6	−710 ± 8	−708 ± 9
*E*_prot_ (mVSCE)	−793 ± 13	−785 ± 7	−785 ±9	−789 ± 7	−790 ± 8
*i*_corr_ (Acm^−2^)	22.5 ± 2.9	9.7 ± 0.7	11.2 ± 0.4	11.6 ± 0.5	12.2 ± 1.1
*P*_EF_ (%)	/	57	50	49	46
*E*_sw_–*E*_corr_ (mV)	25 ± 5	50 ± 2	38 ± 7	38 ± 6	40 ± 2
*E*_corr_–*E*_prot_ (mV)	28 ± 2	35 ± 2	33 ± 4	40 ± 7	46 ± 3
